# Analyses of the pericyte transcriptome in ischemic skeletal muscles

**DOI:** 10.1186/s13287-021-02247-3

**Published:** 2021-03-16

**Authors:** Yuan-chi Teng, Alfredo Leonardo Porfírio-Sousa, Giulia Magri Ribeiro, Marcela Corso Arend, Lindolfo da Silva Meirelles, Elizabeth Suchi Chen, Daniela Santoro Rosa, Sang Won Han

**Affiliations:** 1 Department of Biophysics, Escola Paulista de Medicina, Federal University of São Paulo, Rua Mirassol 207, São Paulo, SP 04044-010 Brazil; 2 Department of Zoology, University of São Paulo, São Paulo, Brazil; 3 Laboratory for Stem Cells and Tissue Engineering, Lutheran University of Brazil, Canoas, Brazil; 4 Department of Morphology and Genetics, Federal University of São Paulo, São Paulo, Brazil; 5 Department of Microbiology, Immunology and Parasitology, Federal University of São Paulo, São Paulo, Brazil; 6 Interdisciplinary Center for Gene Therapy, Federal University of São Paulo, São Paulo, Brazil

**Keywords:** Peripheral arterial disease, Limb ischemia, Muscle, Pericytes, RNA-seq

## Abstract

**Background:**

Peripheral arterial disease (PAD) affects millions of people and compromises quality of life. Critical limb ischemia (CLI), which is the most advanced stage of PAD, can cause nonhealing ulcers and strong chronic pain, and it shortens the patients’ life expectancy. Cell-based angiogenic therapies are becoming a real therapeutic approach to treat CLI. Pericytes are cells that surround vascular endothelial cells to reinforce vessel integrity and regulate local blood pressure and metabolism. In the past decade, researchers also found that pericytes may function as stem or progenitor cells in the body, showing the potential to differentiate into several cell types. We investigated the gene expression profiles of pericytes during the early stages of limb ischemia, as well as the alterations in pericyte subpopulations to better understand the behavior of pericytes under ischemic conditions.

**Methods:**

In this study, we used a hindlimb ischemia model to mimic CLI in C57/BL6 mice and explore the role of pericytes in regeneration. To this end, muscle pericytes were isolated at different time points after the induction of ischemia. The phenotypes and transcriptomic profiles of the pericytes isolated at these discrete time points were assessed using flow cytometry and RNA sequencing.

**Results:**

Ischemia triggered proliferation and migration and upregulated the expression of myogenesis-related transcripts in pericytes. Furthermore, the transcriptomic analysis also revealed that pericytes induce or upregulate the expression of a number of cytokines with effects on endothelial cells, leukocyte chemoattraction, or the activation of inflammatory cells.

**Conclusions:**

Our findings provide a database that will improve our understanding of skeletal muscle pericyte biology under ischemic conditions, which may be useful for the development of novel pericyte-based cell and gene therapies.

**Supplementary Information:**

The online version contains supplementary material available at 10.1186/s13287-021-02247-3.

## Introduction

Pericytes are cells that surround endothelial cells in the vascular system [1–3] and regulate the permeability, stability, and contractility of blood vessels [4, 5]. The relationship between pericytes and endothelial cells is extremely intimate and has been observed in both microanatomy and function. Pericytes extend their processes over endothelial cells, forming “peg-and-socket” contacts [6]. The pericyte coverage of blood vessels estimated by the ratio of pericytes to endothelial cells varies in different tissues according to their physiological function, and it may also be linked to pathological conditions [4, 5, 7]. The interaction between pericytes and endothelial cells also includes ligand-receptor mediated cellular signaling and plays an important role in angiogenesis [1].

In angiogenesis that occurs during embryonic development, pericytes are recruited by endothelial cells via the PDGFRβ/PDGFβ, angiopoietin 1 (Ang1)/Tie2 and transforming growth factor-β (TGFβ) signaling pathways [1]. The molecular mechanisms that underlie neovascularization and pericyte recruitment are well described in early developmental stages, but not in adulthood. PDGFβ/PDGFRβ signaling also participates in this process [1]; however, its role, function, and interplay in various pathological conditions are not fully defined.

Pericytes display a heterogeneous morphology, anatomy, and gene expression profile among different tissues [1, 8, 9]. Additionally, the expression of known pericyte markers can vary, depending on the tissue localization and the zonal effect of vascular structure (artery, capillary, or vein) [1]. These markers are also transiently regulated by the physiological or pathological microenvironment [1]. Consequently, a unique marker specific for pericytes is still lacking. Nonetheless, some markers have been used to indicate a pericyte nature, including PDGFRβ, Nestin, NG2, and CD146 [1]. Interestingly, these and other pericyte markers are also expressed in mesenchymal stem/stromal cells (MSCs), which are widely used in clinical trials to treat various conditions due to their ability to differentiate into mature mesenchymal cells and secrete trophic and immunomodulatory molecules [10, 11]. In vitro, pericytes differentiate into several cell types, such as fibroblasts, adipocytes, muscle cells, Schwann cells, and osteocytes [12–16]. While numerous studies have suggested that pericytes may give rise to other cell types in vivo [17], researchers have debated whether pericytes are multipotent in situ, as contradictory evidence is also available [18]. Similar to MSCs, cultured pericytes modulate the behavior of immune cells [19, 20], and pericytes are also involved in leukocyte recruitment in inflamed tissues [1]. In view of their similarities to MSCs [2, 7], pericytes are promising candidates for cellular therapies, particularly considering their important role in angiogenesis. Successful wound healing usually requires a well-regenerated vascular system with the protection and/or maintenance of pericytes to transport nutrients and oxygen [1]. In addition, several successful therapies using pericytes have been described in animal models of muscle dystrophy [14, 21–23] and skin wounds [24].

Critical limb ischemia (CLI) is the most advanced stage of PAD [25–28]. This illness is mainly caused by atherosclerosis and an increase vessel resistance, which induces an impairment in distal limb perfusion [1]. Patients with CLI suffer from unbearable pain and nonhealing ulcers that may result in lower limb amputation, particularly in individuals with diabetes. Half of patients with CLI cannot be treated by endovascular surgery due to disabilities and other factors [29]. The pathophysiology of CLI is very complex because the advanced stage is characterized by many risk factors, such as hyperlipidemia, hypertension, diabetes, aging, and chronic inflammation. Therefore, an investigation of the pathogenesis of muscle ischemia in a more extensive manner is required to obtain information. This approach can be used to regenerate the vasculature and restore the functionality of the affected muscles.

Previous studies conducted in our laboratory [30–34] and by other researchers [35, 36] have shown that monocytes, macrophages, and MSCs have the potential to regulate angiogenesis, myogenesis, and fibrogenesis in skeletal muscle; therefore, the cells and/or genes that modulate these processes may provide insight into limb ischemia treatment. The role of pericytes in the recovery process from limb ischemia, however, has not been extensively studied. The gene expression profile of pericytes on an omics scale under normal physiological conditions or in pathogenic states has rarely been explored. In particular, the transcriptome of muscle pericytes under ischemic conditions in vivo has not been evaluated to date. Hence, further investigation of the behavior of pericytes during the regeneration of ischemic muscle is warranted.

In skeletal muscles, pericytes are classified into two subpopulations, namely, type I (Nestin^−^) and type II (Nestin^+^). Type I and type II pericytes contribute differently to muscle regeneration, as type II pericytes have myogenic potential and type I pericytes are adipogenic [1]. Pericyte transplantation ameliorates the loss of muscle mass in disease models [12, 14, 21–23]. The possible underlying mechanisms by which pericytes contribute to tissue repair in ischemic muscles include regeneration of myocytes and an improvement in capillarization [12, 21–23].

In this study, we aimed to investigate the transcriptomics of pericytes during the early stages of the regeneration process in a mouse model of surgery-induced hind limb ischemia. In addition, we also aimed to evaluate the frequency of the pericyte subpopulations in muscle throughout the course of ischemia. The changes in gene expression patterns in pericytes before and during the early stages of ischemia are reported here.

## Methods

### Animals

All animal procedures described in this study were approved by the Institutional Animal Care and Use Committee (IACUC) (#6826170118) and performed in full compliance with the recommendations of Federal Law 11.794 (2008), the Guide for the Care and Use of Laboratory Animals of the Brazilian National Council of Animal Experimentation (CONCEA), and the ARRIVE guidelines.

Ten- to 12-week-old male C57BL/6 mice were anesthetized with an intraperitoneal injection of 100 μl of anesthetics (2.5% ketamine/0.26% xylazine in saline) prior to surgery-induced hind limb ischemia. Then, the left femoral artery was exposed and cauterized at its origin between the external iliac artery branch and its bifurcation into the saphenous and popliteal arteries [37] on day 0. The blood flow of the limbs was examined using a laser Doppler imaging (LDI) system (moorLDI2-IR; Moor Instruments, Axminster, United Kingdom). Ischemic mice were orally administered tramadol (20 μl of 24 mg/ml) in the morning and evening to manage pain during the first 2 days after surgery. Ischemic gastrocnemius muscles were collected on postsurgery days 2, 4, and 7 to analyze the pericyte population.

### Flow cytometry

The gastrocnemius muscles were collected and immediately digested with 2 ml of 0.2% collagenase (#C0130, Sigma-Aldrich Co., St. Louis, MO, USA) in Dulbecco’s Modified Eagle’s Medium (DMEM) at 37 °C for 40 min. Tryptic action was then halted by adding the same volume (2 ml) of stop solution [50% fetal bovine serum (FBS)/50% DMEM]. Samples were then sequentially passed through 70-μm and 40-μm cell strainers to remove tissue debris. Cells were washed with PBS once. Samples were then incubated with Fc blockers (#14-0161-81, anti-CD16/CD32 antibody; Thermo-Fisher Scientific, Waltham, MA, USA) at a dilution of 1:1000 in 1% FBS/phosphate-buffered saline (PBS) on ice for 10 min and washed once with PBS. Cells were stained with the following antibodies diluted in PBS: anti-CD45-Pacific blue (clone 30-F11, #MCD4528, Thermo-Fisher Scientific, Waltham, MA, USA; 1:100), anti-CD31-PE/Cy7 (clone MEC13.3, #102524, Biolegend, San Diego, CA, USA; 1:100), and anti-CD146-PerCP/Cy5.5 (clone ME-9F1, #102524, Biolegend; 1:50) on ice for 30 min. After washes with PBS, the cells were stained with the fixable viability dye 780 (#565388, BD Pharmingen, San Jose, California, USA) at a dilution of 1:1000 in PBS on ice for 30 min. After another wash, cells were incubated with Fixation/Permeabilization solution (Cytofix/Cytoperm, #554722, BD Pharmingen) for 20 min at room temperature and washed with Perm/Wash buffer (554,723, BD Pharmingen). An anti-Nestin-PE antibody (clone 307,501, #MA5-23574, Thermo-Fisher Scientific, Waltham, MA, USA; 1:10) diluted in Perm/Wash buffer was used to stain the cells on ice for 30 min. After washes with Perm/Wash buffer, the cells were fixed with 1% paraformaldehyde in PBS for 15 min. Then, after a PBS wash, the cells were resuspended in 2% FBS in PBS and incubated at 4 °C overnight. The cell suspensions from each gastrocnemius muscle were analyzed with a BD LSRFortessa flow cytometer (BD, San Jose, CA, USA) after voltage adjustment using unstained and single-color controls to ensure proper compensation and analysis. In addition, fluorescence minus one (FMO) tubes were used to allow proper gate setting. The number of events acquired per sample ranged from 0.3 to 1.2 million. All flow cytometry data were analyzed using FlowJo Cytometry software (v.10; BD Biosciences). For the flow cytometry analysis, an FSC-A vs. SSC-A dot plot was used to exclude cell debris, and an FSC-A vs. FSC-H dot plot was used to exclude cell clumps and doublets. The number of gated singlet cells obtained after this procedure ranged from 163,000 to 772,000 per sample. Live cells were selected as the viable dye-negative population. Only the CD45^−^ population was subjected to further analyses to exclude nonlymphocytic cells. The pericytes were then identified as a CD31^−^CD146^+^ cell population. Next, the type I (Nestin^−^) and type II (Nestin^+^) pericyte subpopulations were gated. Discrimination of positive events using this gating strategy was based on the FMO samples.

### FACS (fluorescence-activated cell sorting)

Each muscle was digested individually as described above in the flow cytometry section. Multiple digested muscles were combined and filtered through cell strainers. The cells were washed once with PBS and once with wash buffer [3% FBS/1:5000 RNaseOUT (#10777019, Thermo-Fisher Scientific) in PBS]. Samples were incubated with Fc blockers (1:1000 diluted in wash buffer) on ice for 10 min and then washed with wash buffer. The cells were stained with the following antibodies (diluted in wash buffer) on ice for 30 min: anti-CD45-Pacific blue (clone 30-F11, #MCD4528, Thermo-Fisher Scientific; 1:100), anti-CD31-PE/Cy7 (clone MEC13.3, #102524, Biolegend; 1:100), and anti-CD146-PerCP/Cy5.5 (clone ME-9F1, #102524, Biolegend; 1:50). The fixable viability dye 780 was used to stain cells at a 1:1000 dilution in PBS on ice for 30 min. The cells were washed with wash buffer, and the samples were resuspended in wash buffer for cell separation using a BD FASCAria II cell sorter. Compensation adjustment was performed using compensation beads (#552843 and #552845, BD Pharmingen), and FMO controls were used to enable proper gate setting. The sorted cells were collected in collection buffer (10% FBS/1:500 RNaseOUT in PBS) and analyzed to confirm the purity.

Pooled muscle cell suspensions from different mice were used for staining and sorting to ensure that the number of sorted cells was sufficient to provide a sufficient amount of RNA required for the transcriptome analyses. In the nonischemia group, 10 gastrocnemius muscles collected from 5 mice were digested and pooled as one cell suspension sample for subsequent staining and sorting because of the low event number of total cells and pericytes. In ischemic muscles, the event numbers of total cells and pericytes were increased; therefore, two digested muscles were pooled into one cell suspension for further staining and cell sorting.

### Total RNA extraction

Cells sorted by FACS were centrifuged at 3000×*g* for 10 min at 4 °C, and the cell pellet was lysed with 300 μl of TRIzol (#15596026, Thermo-Fisher Scientific, Waltham, MA, USA) with pipetting. After mixing with chloroform and centrifugation, the RNA-containing aqueous supernatant was transferred to a new tube. Next, 0.5 μl of 20 μg/μl glycogen (#10814010, Thermo-Fisher Scientific, Waltham, MA, USA), 40 μl of 2 M sodium acetate, and 500 μl of 100% ethanol were sequentially added to facilitate RNA precipitation. This crude RNA extract was stored at − 80 °C. For further purification, this sample underwent a series of clean-up steps, DNaseI treatment, and RNA elution according to the instructions provided with the Ambion RecoverAll Nucleic acid Isolation Kit (#AM1975, Thermo-Fisher Scientific, Waltham, MA, USA). All the RNA samples were stored in LoBind® microcentrifuge tubes (#22431021, Eppendorf AG, Hamburg, Germany).

### Reverse transcription and cDNA amplification

RNA reverse transcription was performed based on the method described by Picelli et al. [38]. Briefly, for each reaction, 2.3 μl of RNA were mixed with Oligo-dT30VN primers and dNTPs, heated at 72 °C for 3 min, and then chilled immediately on ice. TSO primers mixed with reverse transcriptase were added to perform cDNA synthesis. Next, the cDNA templates were amplified by ISPCR primers and high-fidelity DNA polymerase. The following reagents were used: SuperScript™ IV Reverse Transcriptase (#18090050, Thermo-Fisher Scientific, Waltham, MA, USA), RNaseOUT (#10777019, Thermo-Fisher Scientific, Waltham, MA, USA), KAPA HiFi HotStart ReadyMixPCR Kit (#KK2602, Kapa Biosystems, USA), 5 M Betaine (#B0300-1VL, Sigma-Aldrich Co., St. Louis, MO, USA), and 1 M MgCl_2_ (#M1028-10X1ML, Sigma-Aldrich Co., St. Louis, MO, USA). AMPure XP magnetic beads (#A63880, Beckman Coulter, Brea, CA, USA) were used to purify the cDNAs. The concentrations of cDNAs were then determined using Qubit DNA HS assay (#Q32854, Thermo-Fisher Scientific, Waltham, MA, USA).

### RNA-seq library preparation and data analysis

The Nextera XT DNA Library Prep Kit was used to construct cDNA libraries. Sequencing was performed using a NextSeq 500/550 High-Output v2.5 Kit (150 cycles) (#20024907, Illumina, San Diego, CA, USA) at CEFAP GENIAL (Genome Investigation and Analysis Laboratory; http://cefap.icb.usp.br/core-facilities/genial-genome-investigation-and-analysis-laboratory/). Raw data were first examined using FastQC (Version 0.11.8). After trimming with Trimmomatic (Version 0.39) [39] using the default settings, the data were analyzed with FastQC again. Rsubread (Version 1.28.1/R Version 3.4) was used for both alignment and generation of counts [40]. The count per gene database was then inputted to DESeq2 (Version 1.24.0) for differential expression analyses [41]. The time-course pattern analysis was performed using the Short Time-series Expression Miner (STEM) program [42]. Statistically significant differences reported by STEM are based on a correlation test at a significance level of 0.05 followed by Bonferroni correction of the *p* value. The GO enrichment analysis was conducted using the software package “ClusterProfiler” version 3.12.0 [43]. Heatmaps were generated using the software package “pheatmap” using rlog transformed counts calculated by “DEseq2”.

## Results

### Recovery of blood flow after surgery-induced ischemia

**Fig. 1:**
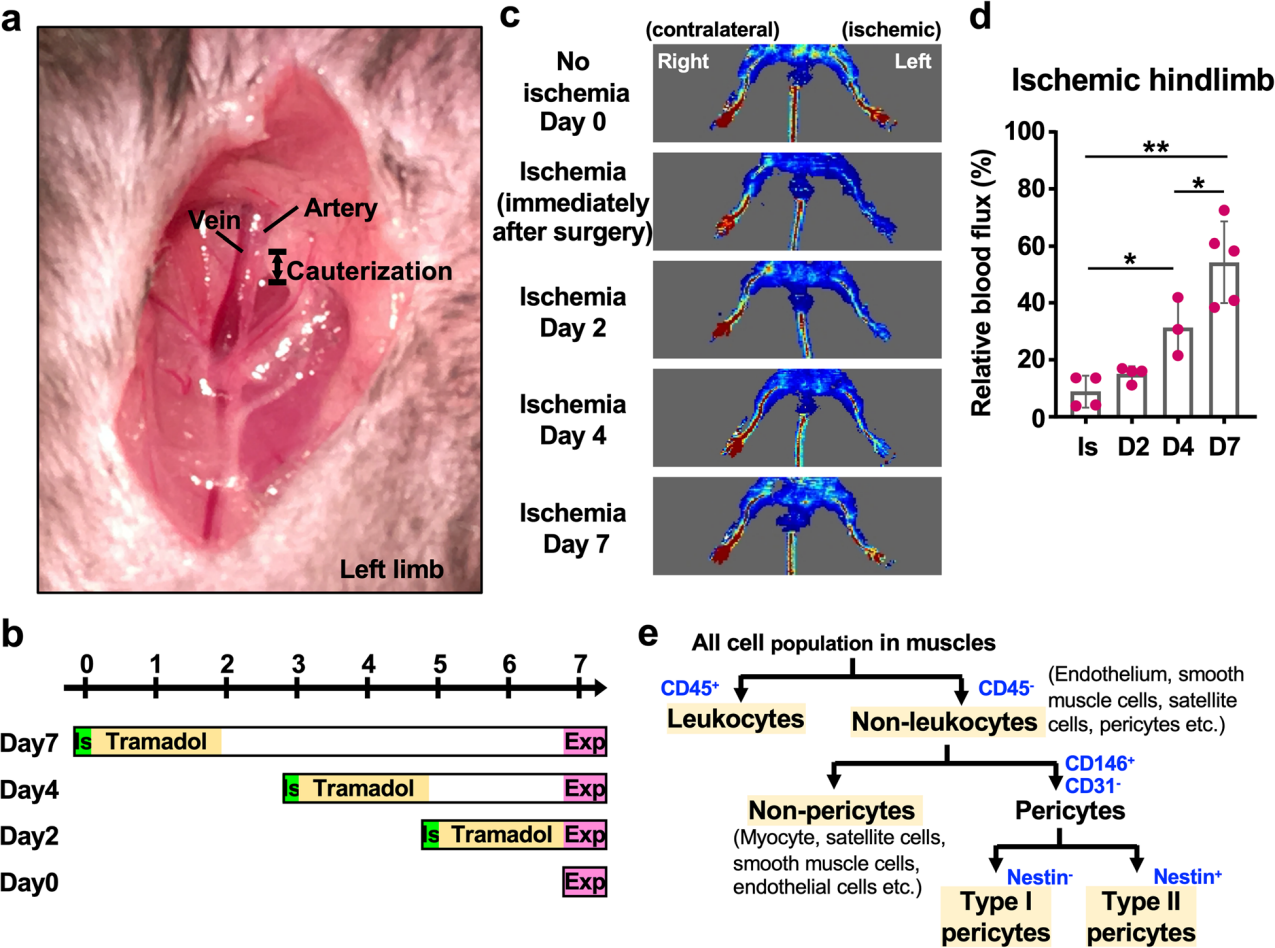
Analysis of pericyte biology in a mouse model of hindlimb ischemia. **a** Surgery to induce hindlimb ischemia was performed on the left leg of the mouse. After dissociating the artery from the vein, a segment of the artery was electrocauterized. **b** Schematic graph of the timeline for sample collection after ischemia surgery. IS, ischemia; Exp, experiment (i.e., flow cytometry and FACS). **c** The Doppler images show the blood flow in the ischemic and contralateral limbs at 0, 2, 4, and 7 days after surgery. The red signal represents the abundant blood flow. **d** The quantitation of blood flux of ischemic hindlimbs (left) relative to contralateral hindlimbs (right). The measurement was performed by MoorLDI software (version 5.2, Moore Instruments, Axminster, UK). *Student’s t* test was used to perform statistical analyses (*p < 0.05; **p < 0.01). IS, immediately after ischemia surgery. The mouse numbers used for Doppler scanning on ischemia day 0, 2, 4, and 7 were 4, 4, 3, and 5, respectively. **e** The strategy used to identify endothelial cell and pericyte populations in flow cytometry and FACS experiments

We first surgically induced ischemia in the left hind limb of C57BL/6 mice through electrocauterization of the femoral artery to study the role of muscle pericytes in the response to ischemia (Fig. 1a, b). The assessment of blood flow using LDI showed abundant blood flow before surgery and successful disruption of the blood flow of the left limb after surgery (Fig. 1c, d). No signs of darkening nails or gangrene were observed in the C57BL/6 mice after surgery, as assessed by a daily visual inspection throughout the experimental period (maximal 7 days). Mice presented a slight limp on the ischemic limb only during the first 2–3 days, but the limp recovered later. The appearance and physical activity of mice on day 7 postischemia were similar to the animals in the nonischemic group. The LDI examination showed that blood flow was gradually restored (Fig. 1c, d). Notably, the blood flow was partially reestablished in the left digits 7 days after surgery-induced ischemia (54.2 ± 14.4%) (Fig. 1c, d).

### Ischemia induces the expansion of the pericyte population

Endothelial cells and pericytes are the main components of blood vessels and work together to maintain vascular homeostasis, particularly after injury. Thus, variations in the numbers of these cells, including subtypes of pericytes in muscle [12, 13], were initially evaluated at different time points after ischemia to study the effect of ischemia on vascular homeostasis. As ischemia affects tissues distant from the obstructed region, we focused on the gastrocnemius muscle for analysis using flow cytometry.

CD146, PDGFRβ, and NG2 are the most commonly used markers to identify pericytes for cell sorting. However, our attempts to discriminate pericytes based on the detection of PDGFRβ and NG2 using antibodies failed to identify a distinctive positive signal for these markers compared to FMO controls using flow cytometry (data not shown) when we performed pilot tests. Failure to detect NG2 might be related to the low expression of this marker in the absence of injury, as surface expression of this marker in freshly isolated resting human pericytes is very low and difficult to detect using flow cytometry [44]. We speculate that the inability to distinctively discriminate PDGFRβ^+^ cells using flow cytometry is related to the relatively low expression of this marker with a high fluorescence background under the conditions used for immunostaining. Consequently, we opted to use CD146 as a marker to discriminate and isolate pericytes. Furthermore, we performed intracellular immunostaining to assess Nestin expression in cells from wild-type mice. In the flow cytometry analysis, the leukocyte population was distinguished by the CD45 hematopoietic marker. The remaining CD45^−^ cells are composed of mainly endothelial cells, smooth muscle cells, fibroblasts, satellite cells, and pericytes. Using antibodies against CD31 and CD146, endothelial cells were identified as CD31^+^CD146^−^ cells, while pericytes were identified as CD31^−^CD146^+^ cells. Pericytes were further subdivided into type I (Nestin^−^) and II (Nestin^+^) subpopulations (Fig. 1d).

**Fig. 2:**
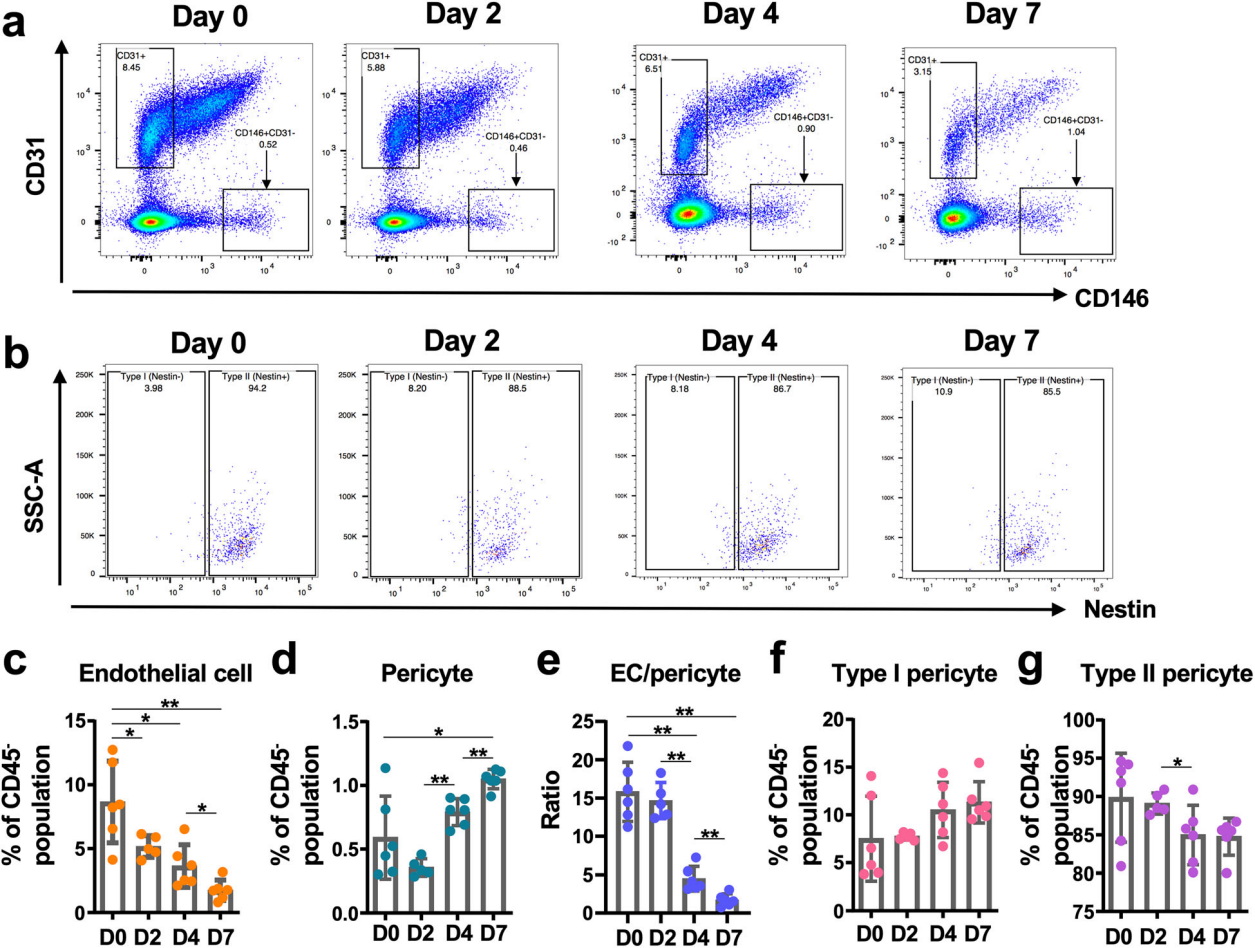
Dynamics of endothelial cell and pericyte populations upon ischemia. **a** Representative images of endothelial cell (CD31^+^CD146^−^) and pericyte (CD146^+^CD31^−^) populations in gated CD45^−^ populations from ischemic gastrocnemius and soleus muscles. **b** Representative flow cytometry images of subtypes of pericytes based on Nestin expression. **c** Quantitation of the endothelial cell population in the CD45^−^ population from ischemic muscles. **d** Quantitation of the pericyte population in the CD45^−^ population from ischemic muscles. **e** The ratio of endothelial cell (EC) events/pericyte events. **f**, **g** The ratios of type I (**f**) and type II (**g**) pericytes in pericyte populations. *Student*’s *t* test was used to perform statistical analyses of the data presented in **c**–**g**. *p < 0.05; **p < 0.01. The numbers of mice used for flow cytometry on ischemia D0, D2, D4, and D7 were 6, 5, 6, and 6, respectively

In muscles without ischemia (Day 0; hereafter D0), the frequencies of endothelial cells (CD31^+^CD146^−^) and pericytes (CD31^−^CD146^+^) were quantified as 8.6 ± 3.2% and 0.6 ± 0.3% of the CD45^−^ population, respectively (Fig. 2a–d)*.* Therefore, the ratio of endothelial cells to pericytes was approximately 15:1 in skeletal muscle under normal physiological conditions (Fig. 2e). Upon the induction of ischemia, the ratio of CD31^+^ endothelial cells to pericytes in the CD45^−^ population gradually decreased until the last time point (7 days postischemia) (Fig. 2c); conversely, the total pericyte population increased rapidly and continuously after day 2 (Fig. 2d)*.* The number of events corresponding to endothelial cells and pericytes per sample analyzed using flow cytometry also clearly showed the dramatic alteration in cell number in the ischemic muscles (Supplementary Figure 1A and B). The dynamic alterations in both cell types in the opposite direction were noted by the endothelial cell/pericyte ratio on days 4 and 7 postischemia, which decreased to 4.5 and 1.7, respectively (Fig. 2e). Under nonischemic conditions (D0), type II pericytes (Nestin^+^CD31^−^CD146^+^) were predominant in skeletal muscle, corresponding to approximately 90% pericytes. After ischemia, the prevalence of type II pericytes slowly decreased, reaching 84.8% on day 7 (Fig. 2g). This event was compensated by a proportional increase in the type I pericyte population (Nestin^−^CD31^−^CD146^+^) (Fig. 2f, g). Taken together, these findings indicate that the pericyte population expanded after ischemia, while the ratio of type I to type II pericytes remained similar.

### Ischemia-induced differentially expressed genes

**Fig. 3:**
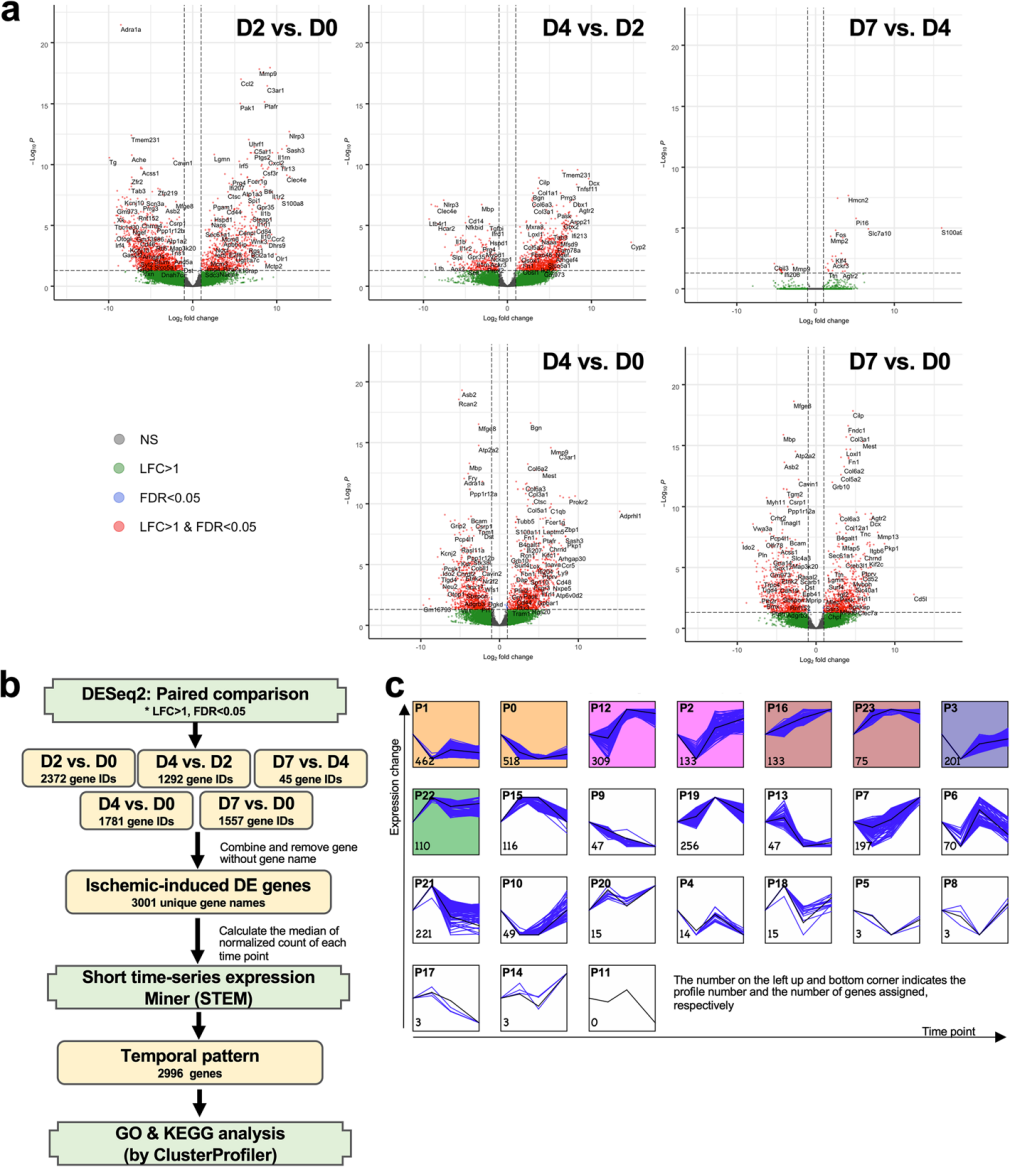
Differentially expressed genes in response to ischemia in pericytes. **a** The volcano plots show the DE genes with a log fold change (LFC) > 1 and/or false discovery rate (FDR) < 0.05. **b** The strategy used to analyze the dynamics of DE genes at different time points after ischemia. **c** The result of STEM analyses. The profile number and assigned gene number are indicated at the top left and bottom corners, respectively. The number of RNA samples/libraries used for the RNA-seq analysis at ischemia D0, D2, D4, and D7 was 6, 5, 8, and 8, respectively

Pericytes enzymatically dissociated from muscles were stained with anti-CD45, CD146, and CD31 antibodies, and then CD45^−^CD146^+^CD31^−^ cells were isolated by FACS on days 0, 2, 4, or 7 postischemia to assess the transcriptional regulation in muscle pericytes during the early phases of ischemia (Supplementary Figure 2). RNA from these sorted pericytes was subjected to an RNA-seq analysis. The differentially expressed genes (DEGs) in each comparison were further illustrated using volcano plots (Fig. 3a). The greatest alteration in the transcriptome of pericytes revealed 2372 DEGs (Ensembl gene IDs) in the ischemia group on day 2 compared with the nonischemia group (D0) (Fig. 3a). A comparison between ischemic days 2 (D2) and 4 (D4) showed that 1292 DEGs were differentially expressed in pericytes of ischemic muscles (Fig. 3a). However, the number of DEGs identified from day 4 (D4) to day 7 (D7) was much lower, showing that the effect of ischemia on pericytes occurs during the first 4 days postischemia. Only 45 Ensembl gene IDs were defined as DEGs by DESeq2 (Fig. 3a). Nonetheless, either at day 4 or at day 7, pericytes expressed a large number of DEGs compared to the nonischemia group (Fig. 3a). Since we were interested in the dynamic change in DEGs from days 0 to 7, the whole set of DEGs from all paired comparisons was applied to STEM (short time-series miner) [42] for pattern analyses (Fig. 3b). The 2996 DEGs were divided into 23 patterns, which are called profiles hereafter (Fig. 3c).

**Fig. 4:**
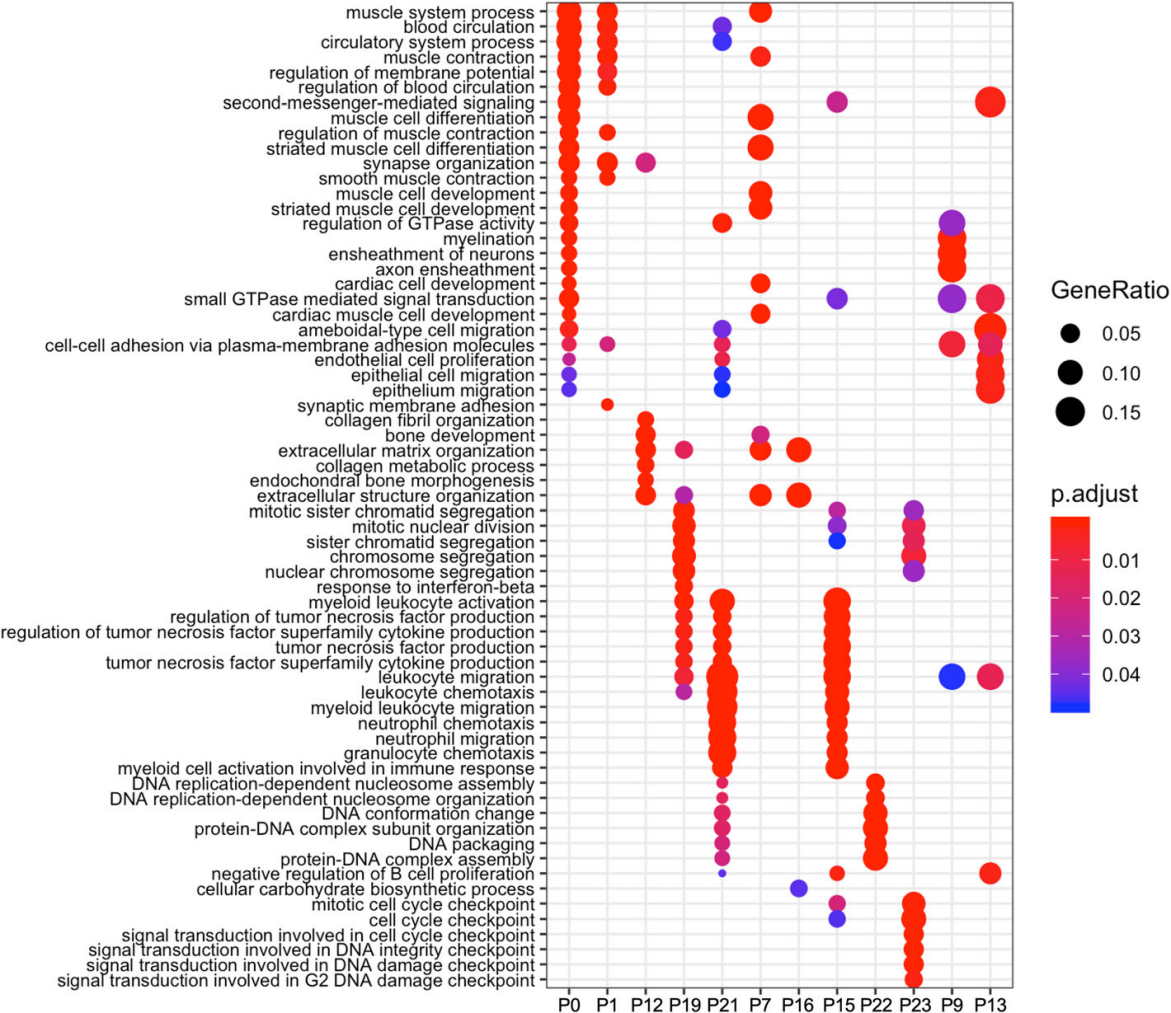
Comparison of enriched GO categories between dynamic profiles. The results of analyses and comparisons of the enriched GO terms in each dynamic profile determined by STEM using ClusterProfiler packages

GO (Gene Ontology) and KEGG enrichment analyses were further conducted on an individual profile containing more than 40 DEGs to understand whether a single profile has a specific biological meaning (Fig. 3b). The GO terms for the biological process category in each profile are summarized in Fig. 4 (see the details in Additional file 1). In our current data, the KEGG enrichment analysis did not provide a better understanding of the biological meaning (Supplementary Figure 3). For profiles 1 and 0, which have a similar pattern, the GO terms of DEGs were mainly involved in the muscular system and blood circulation, suggesting that the genes involved in these two processes were dramatically downregulated at day 2 and their expression levels remained low until day 7 (Fig. 4). DEGs in profile 12 and profile 16 were enriched for the GO term organization of extracellular matrix, implying the expression of genes related to tissue recovery from ischemia (Fig. 4). The GO analysis of profile 19 indicated the existence of active cell division and cytokine regulation peaking at day 4 (Fig. 4). GO categories involving cytokine production were observed in profile 21, which exhibited maximal expression at day 2, followed by a reduction (Fig. 4). Profile 15 contained DEGs that also played a role in the regulation of cytokines and the cell cycle (Fig. 4). Nevertheless, most of these genes were expressed maximally between day 2 and day 4*.* DEGs in profile 7 were mostly involved in the regulation of the muscle system and extracellular matrix composition. These genes were gradually upregulated over time under ischemic conditions (Fig. 4). In profiles 22 and 23, the GO enrichment analysis showed a significant enrichment of genes involved in the cell cycle, which were upregulated from day 2 to day 7 (Fig. 4). A consistent decrease in the number of DEGs playing a role in myelination was observed in profile 9 (Fig. 4), consistent with a recent report showing that pericytes are essential for oligodendrocyte progenitor differentiation and myelin formation during remyelination in the brain [45]. Consequently, profile 9 suggests the occurrence of demyelination during the first week after ischemia. DEGs in profile 13 were related to endothelial cell proliferation or epithelial cell migration and were downregulated from day 4 to day 7 after induction of ischemic injury (Fig. 4). This profile corroborates a decrease in the number of endothelial cells in the ischemic muscle at these experimental time points (Fig. 2c and Supplementary Figure 1a).

### Ischemia-induced genes in pericytes are related to myogenesis

**Fig. 5:**
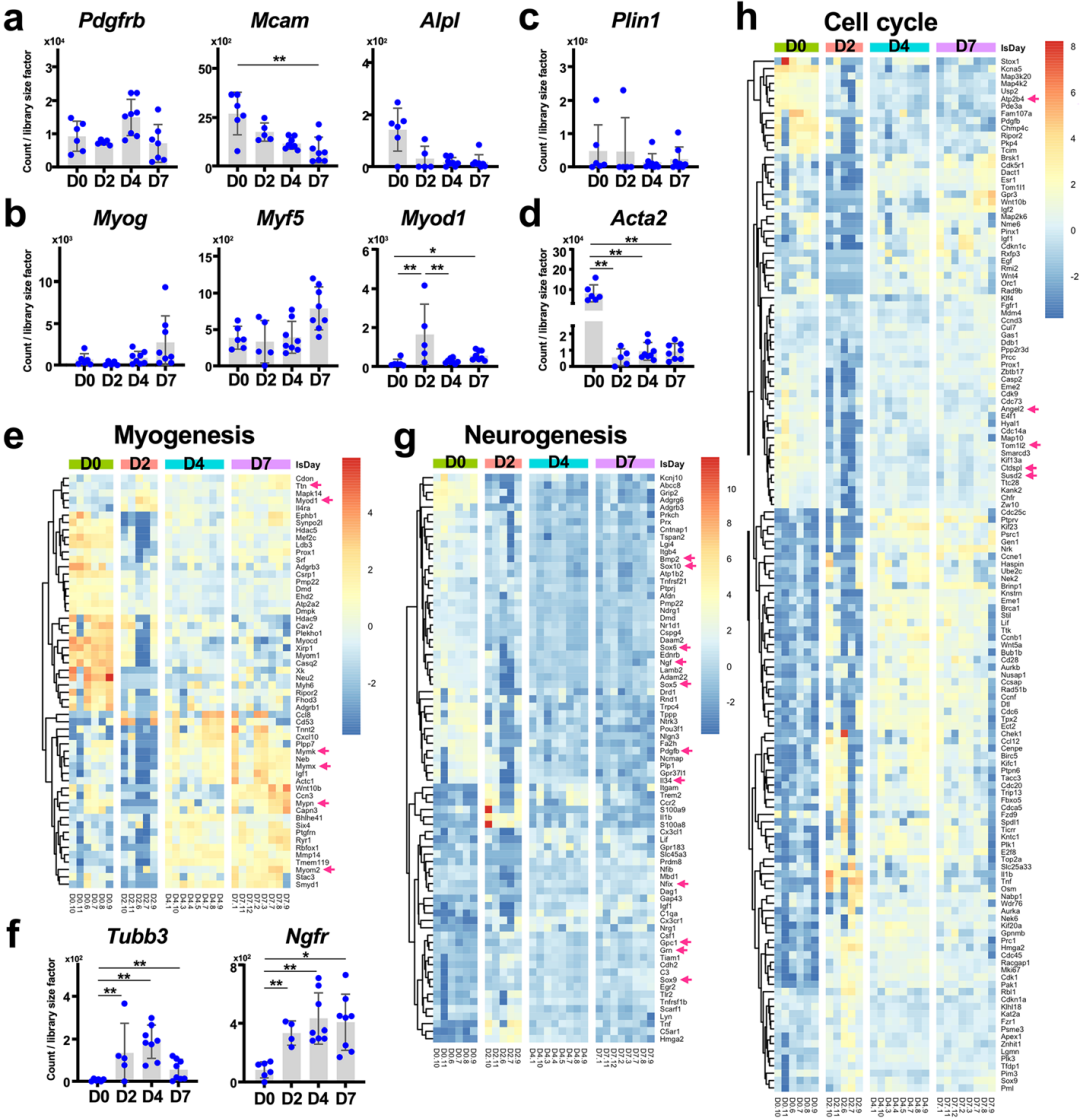
Gene expression profiles involved in myogenesis, neurogenesis and the cell cycle in ischemic pericytes. **a** The expression profiles of pericyte markers. **b** The expression levels of myogenic genes in ischemic pericytes. **c** The expression level of *Plin1*, an adipogenic marker, in ischemic pericytes. **d** The expression level of *Acta2*, a fibrogenic gene, in ischemic pericytes. **e** A heatmap presenting the expression profiles of genes involved in muscle development and structure according to GO trees (see the details in Table 1). **f** The expression levels of neurogenic markers in ischemic pericytes. **g**, **h** Heatmaps presenting the expression profiles of genes involved in neurogenesis (**g**) and cell cycle regulation (**h**) according to GO trees (see the details in Table 1). The results of the statistical analysis shown in (**a–d**, **f**) are the FDR calculated using Deseq2. *FDR < 0.05; **FDR < 0.01. The genes mentioned in the text are marked with arrows

We first examined the expression of known pericyte, myogenic, adipogenic, fibrogenic, and neurogenic markers to address the multipotent differentiation potential of pericytes. Genes that encode the pericyte markers CD146 (*Mcam*) and alkaline phosphatase (*Alpl*) displayed a decreasing trend, which was not observed for *Pdgfrb* (which encodes PDGFRβ; CD140b) (Fig. 5a). Interestingly, we observed increased expression of myogenic genes, including *Myog*, *Myf5*, and *Myod1* (Fig. 5b, e), although only *Myod1* expression exhibited a statistically significant difference, with peak expression at ischemia day 2. Importantly, these expression profiles were similar to those reported in a previous study of human pericytes [1]. We further selected the genes related to muscle development and structure according to the GO tree or term (see the details in Table 1) and plotted a heatmap of DEGs to reveal the relative expression levels among samples. We observed a number of upregulated genes related to myocyte maturation, such as *Ttn*, *Mymk*, *Mymx*, *Mypn*, and *Myom2*, at ischemia day 7 (Fig. 5e). Furthermore, some downregulated DE genes, such as *Adgrb3* and *Hdac9*, were observed, which are known to regulate other biological processes (Fig. 5e).

**Table 1:** Classification of biological functions in heatmaps according to GO categories

Name	GO ID	GO name
**Skeletal muscle development (**Fig. 5e**)**	1902725	Negative regulation of satellite cell differentiation
	1902810	Negative regulation of skeletal muscle fiber differentiation
	0048632	Negative regulation of skeletal muscle tissue growth
	1902811	Positive regulation of skeletal muscle fiber differentiation
	0048743	Positive regulation of skeletal muscle fiber development
	0048633	Positive regulation of skeletal muscle tissue growth
	2001014	Regulation of skeletal muscle cell differentiation
	0048742	Regulation of skeletal muscle fiber development
	0048631	Regulation of skeletal muscle tissue growth
	0048741	Skeletal muscle fiber development
	0045214	Sarcomere organization
	0048769	Sarcomerogenesis
	0030241	Skeletal muscle myosin thick filament assembly
	0033292	T-tubule organization
	0007520	Myoblast fusion
	0014904	Myotube cell development
	0014908	Myotube differentiation involved in skeletal muscle regeneration
	0010832	Negative regulation of myotube differentiation
	0010831	Positive regulation of myotube differentiation
	0010830	Regulation of myotube differentiation
	0098528	Skeletal muscle fiber differentiation
	1901740	Negative regulation of myoblast fusion
	0060299	Negative regulation of sarcomere organization
	0060298	Positive regulation of sarcomere organization
	0060297	Regulation of sarcomere organization
	0014866	Skeletal myofibril assembly
**Neurogenesis (**Fig. 5g**)**	0014013	Regulation of gliogenesis
	0010001	Glial cell differentiation
	0016322	Neuron remodeling
	0014041	Regulation of neuron maturation
**Cell cycle (**Fig. 5h**)**	0010564	Regulation of cell cycle process
	1901976	Regulation of cell cycle checkpoint
	0007346	Regulation of mitotic cell cycle
**Vasculogenesis (**Fig. 6a**)**	0035441	Cell migration involved in vasculogenesis
	2001212	Regulation of vasculogenesis
	0060312	Regulation of blood vessel remodeling
	1904752	Regulation of vascular associated smooth muscle cell migration
	1901432	Regulation of vasculature development
	1990936	Vascular smooth muscle cell dedifferentiation
	1905651	Regulation of artery morphogenesis
	1904753	Negative regulation of vascular associated smooth muscle cell migration
	0045765	Regulation of angiogenesis
	0002040	Sprouting angiogenesis
	0120078	Cell adhesion involved in sprouting angiogenesis
**VEGF (Fig.** **6****b)**	0048010	Vascular endothelial growth factor receptor signaling pathway
**Effect of endothelial cells (Fig.** **6****c)**	1901509	Regulation of endothelial tube morphogenesis
	0045601	Regulation of endothelial cell differentiation
	0001936	Regulation of endothelial cell proliferation
**Response to ischemia (Fig.** **6****d)**	0002931	Response to ischemia
**Cell junction organization (Supplementary Fig.** **3****a)**	0034330	Cell junction organization
**Regulation of cell adhesion (Supplementary Fig.****3****c)**	0030155	Regulation of cell adhesion
**Extracellular matrix (Supplementary Fig.****3****b)**	0030199	Collagen fibril organization
	0085029	Extracellular matrix assembly
	0001952	Regulation of cell-matrix adhesion
**Leukocyte activation (Supplementary Fig.****3****d)**	0045321	Leukocyte activation

In addition, we observed upregulated expression of transcripts encoding some neurogenesis-related markers, such as *Tubb3* and *Ngfr* [1], on day 2 after ischemia (Fig. 5f). Notably, *Tubb3* (neuron-specific β-tubulin) expression was not constantly maintained after induction by ischemia. *Tubb3* expression has been shown to be a marker of activated, proangiogenic pericytes, in addition to its role in neuronal cells [46]. Likewise, upregulation of NGFR (CD271) has been associated with pericyte activation in other tissues, such as the liver [47] and heart [48]. Therefore, we decided to explore a possible association of pericytes with neurogenesis by generating a heatmap of DEGs that matched the GO term neurogenesis (Fig. 5g and Table 1). Consequently, the degree of matching DEGs was less obvious than for the GO term myogenesis. The generated heatmap showed that the expression of *Grn*, *Gpc1*, and *Nfix*, whose products are involved in nerve function, was induced in pericytes (Fig. 5g). Additionally, we observed the downregulation of transcripts encoding a series of secretory factors (such as *Ngf*, *Bmp2*, *Pdgfb*, and *Il34*) and transcription factors (Sox family) that control a broad range of cellular activities (Fig. 5g).

Our RNA-seq data also showed that the expression levels of *Plin1* (Perilipin 1), an adipogenic marker, were relatively low and remained unchanged after ischemia (Fig. 5c). Additionally, the expression of *Acta2* (which encodes α-smooth muscle, α-SMA) was dramatically abolished in pericytes under ischemic conditions (Fig. 5d). Based on these results, pericytes are less committed to adipogenesis and fibrogenesis in ischemic muscles.

Consistent with the phenomenon of pericyte expansion (Fig. 2d and Supplementary Figure 1b), GO enrichment analyses revealed a significant upregulation of cell cycle-related genes (Figs. 4 and 5h). Induced genes, such as cyclins and cyclin kinases, were observed from ischemia days 4 to 7 (Fig. 5h). Additionally, the expression of some negative regulators of the cell cycle, such as *Atp2b4*, *Ctdspl*, *Susd2*, *Angel2*, and *Tom1 l2*, was significantly diminished after ischemia day 2 (Fig. 5h). Thus, pericytes actively proliferate after ischemia.

### Transcriptome of pericytes during vascular regeneration

**Fig. 6:**
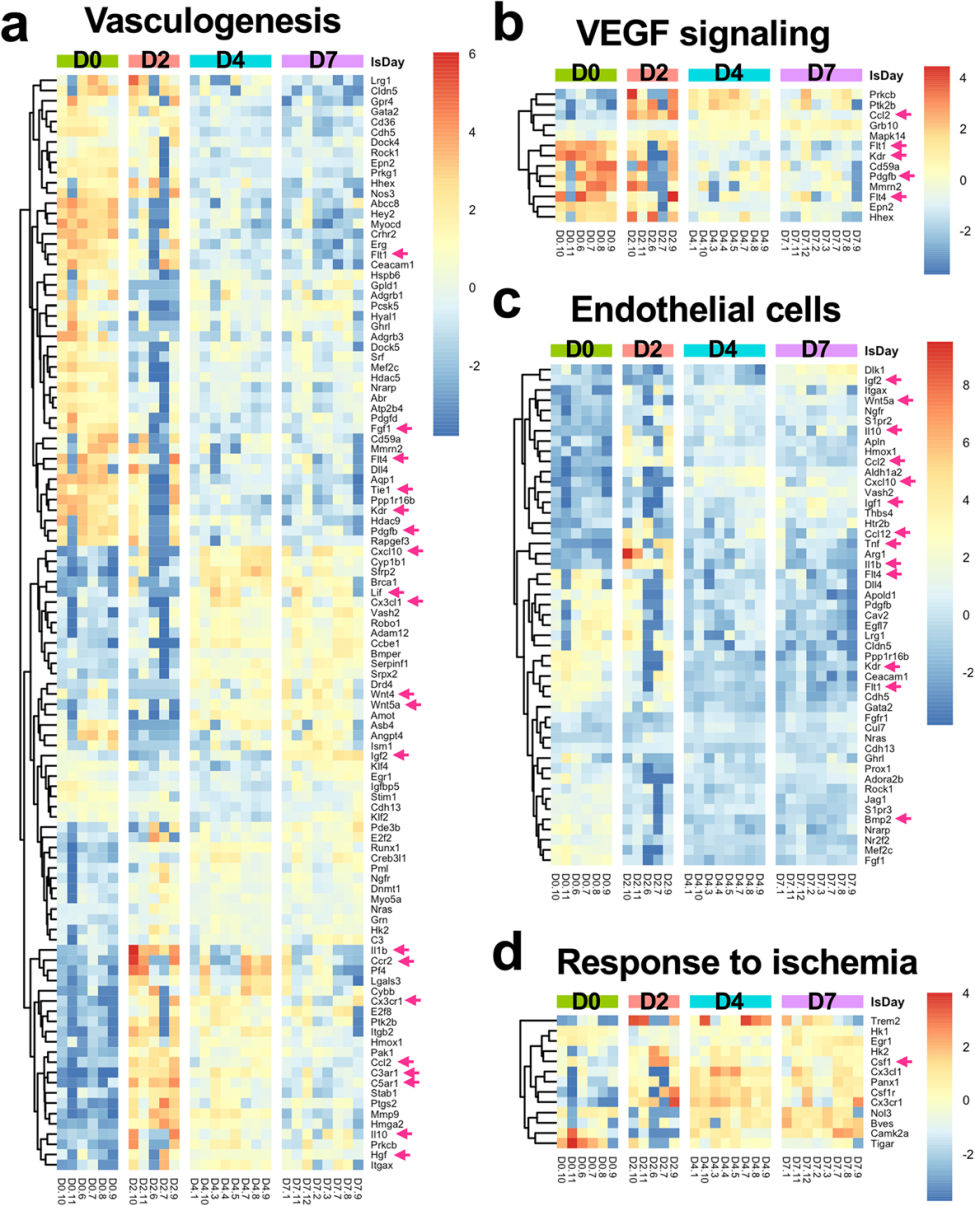
Heatmaps of expression levels in the vascular system. Heatmaps present the expression profiles of genes involved in **a** vasculogenesis and angiogenesis, **b** VEGF signaling, **c** regulation of endothelial cells, and **d** response to ischemia. The genes listed here were manually selected according to GO trees (see the details in Table 1). The genes mentioned in the text are marked with arrows

Next, we analyzed genes involved in vasculogenesis, angiogenesis, and ischemia, which are essential processes for the repair and regeneration of ischemic muscle. As in the previous analyses, we selected DEGs from GO terms related to vasculogenesis, angiogenesis, VEGF signaling, and regulation of endothelial cells, and then evaluated their expression levels in our RNA-seq data (see Table 1). The mRNA expression levels of cytokines and cytokine receptors were significantly altered over time after ischemia. Notably, the transcripts of all VEGF receptors, namely, *Flt1* (VEGFR1), *Kdr* (VEGFR2), and *Flt4* (VEGFR3), were significantly downregulated after ischemia (Fig. 6a–c). In addition, decreased expression levels of transcripts encoding cytokines such as *Tie1*, *Pdfgb*, and *Fgf1* were also observed (Fig. 6a). Two days after ischemia induction, the expression of cytokine genes, such as *Ilb1*, *Ccl2*, *Il10*, and *Hgf*, was induced (Fig. 6a). These genes were continuously expressed in pericytes at ischemia day 4, but their levels were reduced at day 7. The cytokine receptor genes *Ccr2*, *C3ar1*, *Cx3cr1*, and *C5ar1* were expressed in a similar dynamic pattern as *Ilb1* (Fig. 6a). Later, at ischemia day 4, another wave of upregulation of cytokine genes (*Cxcl10*, *Lif*, *Cx3cl1*, *Wnt4*, and *Wnt5a*) occurred, lasting until day 7 (Fig. 6a). One particular cytokine gene, *Igf2*, was induced only at ischemia day 7 (Fig. 6a). Among the aforementioned cytokines and cytokine receptor genes, *Ccl2*, *Pdgfb*, *Flt1*, *Kdr*, and *Flt4* are known regulators of VEGF signaling (Fig. 6b).

The tight relationship between endothelial cells and pericytes prompted us to explore the genes regulating the differentiation and proliferation of endothelial cells. In addition to the cytokine expression (*Igf1, Igf2*, *Wnt5a*, *Il10*, *Ccl2*, *Cxcl10*, *Il1b*, and *Bmp2*) shown in Fig. 6a, pericytes also expressed the cytokines *Ccl12* and *Tnf* (Fig. 6c), which not only exert effects on endothelial cells after ischemia but are also chemoattractants for monocytes and neutrophils.

During vasculogenesis and angiogenesis, pericytes dissociate from endothelial cells, migrate, and reattach to endothelial cells. The expression of transcripts encoding collagens and matrix metallopeptidases (MMPs) was detected as DEGs in volcano plots (Fig. 3a). Hence, we also examined in detail the DEG expression profiles related to cell junctions, cell adhesion, and extracellular matrix (ECM). First, we observed significant *Snail* upregulation in pericytes after ischemia, indicating a possible tendency of migration (Supplementary Figure 4a). Dynamic changes in the expression profile of the cadherin family were also observed, suggesting that cell junctions are remodeled after ischemic injury. Under nonischemic conditions, pericytes expressed *Cdh19*, *Cdh6*, *Cdh10*, *Cdh4*, *Cdh13*, and *Cdh5*; after ischemia, the cadherin genes that were predominantly expressed were *Cdh15*, *Cdh11*, *Cdh3*, and *Cdh2* (Supplementary Figure 4a). Cdh2 has been described as an important molecule in cell junctions forming peg-and-socket contacts between endothelial cells and pericytes [2, 49]. Dynamic alterations were also observed in the expression of the claudin family, whose members participate in tight junctions; the expression of *Cldn19*, *Cldn5*, and *Cldn1* decreased in pericytes under ischemic conditions.

A similar result was also obtained for genes encoding components of the ECM. The expression of collagen genes was altered during the experiment. *Col11a2*, which was expressed at high levels in pericytes under normal physiological conditions, was substantially downregulated 2 days after ischemic injury and reached appreciable expression levels on days 4 and 7 of the experiment (Supplementary Figure 4b). *Col1a1*, *Col1a2, Col3a1*, *Col5a1*, *Col5a2*, and *Col14a1*, on the other hand, were substantially upregulated at days 4 and 7 after induction of ischemia (Supplementary Figure 4b). Furthermore, the upregulation of *Mmp12* and *Mmp14* was also observed beginning at ischemia day 4 (Supplementary Figure 4b). Additionally, fluctuations in the levels of the transcripts of laminin α and laminin β proteins, which are major components of the basal lamina and include *Lama1*, *Lama3*, *Lama4*, *Lama5*, *Lamb1*, *Lamb2*, and *Lamb3*, were also observed (Supplementary Figure 4b and c). Taken together, these changes in the expression of cadherins and claudins mentioned above may reflect an alteration in the physical contact of pericytes with endothelial cells and basement membrane molecules induced by ischemic injury.

The GO term “response to ischemia” included 38 genes related to an inadequate blood supply. Among these genes, DE ischemia-responsive genes in pericytes covered approximately 34% (13 genes) of the “response to ischemia” group in the GO analysis, and most of these genes were upregulated after 4 days of ischemia (Fig. 6d).

Pericytes possess immunoregulatory properties, particularly in the brain [19, 20, 50, 51]. Pericytes upregulated the expression of signaling molecules such as cytokines (*Il1b*, *Tnf*, and *Il10*) and chemokines (*Ccl3*, *Ccl2*, *Ccl4*, *and Cxcl5*) at day 2 after ischemic injury (Supplementary Figure 5a). Genes encoding three Toll-like receptors (*Tlr8*, *Tlr1*, and *Tlr2*) were slightly upregulated in pericytes at 4 days after the induction of ischemia (Supplementary Figure 5a). Based on these observations, pericytes may modify the behavior of immune cells during muscle ischemia.

## Discussion

### The dynamic responses of endothelial cells and pericytes to ischemia

We generated a limb ischemia model by disrupting the femoral artery and then analyzed the lower part of the limb, the gastrocnemius muscles, to understand the roles of muscle pericytes in the regenerative process that occurs during ischemia. The endothelial cell population in the gastrocnemius muscle decreased after ischemia (Fig. 2a, c and Supplementary Figure 1a). The possible explanation for this phenomenon is that endothelial cells die due to a lack of sufficient oxygen and nutrients. A less likely scenario is that a portion of endothelial cells in the gastrocnemius muscle may migrate to areas of active angiogenesis [1, 4, 7, 8]. On the other hand, the pericyte population increased after ischemia day 4 (Fig. 2a, d and Supplementary Figure 1b). Based on this finding, pericyte proliferation occurs locally, consistent with the observation that pericytes proliferate in response to trauma [52] or hypoxia [7, 8]. Our transcriptomic analyses clearly revealed the upregulation of genes involved in mitotic cell division in pericytes after the induction of ischemia (Figs. 4 and 5h). Additionally, the number of pericytes and endothelial cells, as well as the ratio of endothelial cells to pericytes on ischemia day 7, were still different from those observed under normal physiological conditions (day 0; Fig. 2c–e). Thus, although blood flow was significantly restored, these two cell populations may still be recovering.

An important point of the methodology used to estimate the relative numbers of pericytes and endothelial cells in this study is that these numbers were normalized to the number of CD45^−^ cells in each analysis. Any cell type in the CD45- population other than endothelial cells and pericytes, which include smooth muscle cells, satellite cells, fibroblasts, and other cell types, also has the potential to undergo cell proliferation or death at any time point in an ischemic environment. This approach may have affected the calculation of the populations of endothelial cells and pericytes.

### Limitation of our transcriptomic analyses

Transcriptome analyses of pericytes are currently less available, particularly regarding the discussion of the time-dependent variation of the transcriptome in response to stress or treatment. Here, we report for the first time the dynamic change in the transcriptome of pericytes in the ischemic hindlimb to mimic peripheral arterial disease. Due to some limitations we faced in obtaining and purifying pericytes in large quantities of skeletal muscles, we include the following comments that discuss these issues’ influence on interpreting the data.

Data and analyses of the pericyte transcriptome are currently less available, particularly analyses that discuss changes in the transcriptome in a time-dependent manner in response to stress or treatment. Here, we report for the first time the dynamic change in the transcriptome of pericytes in the mouse ischemic hindlimb to simulate peripheral arterial disease.

In our work, we used a pool of pericytes without separating them into type I and II pericytes to perform RNA-seq analyses because the discrimination of these pericyte subtypes by FACS involves the identification of Nestin expression by intracellular staining, which usually leads to loss of RNA yield and stability due to cell permeabilization. Consequently, the differential gene expression of the type I pericyte in the RNA-seq result is highly likely to be diluted due to the relatively low population of type I pericytes in the muscles (Fig. 2f, g). To improve our study’s scope and resolution, single-cell RNA-seq can be used in future experiments to sub-group type I and II pericytes.

To obtain a single cell suspension from muscle tissue, we used collagenase digestion. The full detachment of pericytes from endothelial cells and basement membrane is difficult to achieve because of the junctions that connect these two cell types, in addition to the density of the basement membrane matrix in which these cells are embedded. Therefore, some degree of contamination of the isolated pericytes with endothelial cells is practically inevitable, particularly on D0. Consequently, we estimated the degree of EC contamination by analyzing the expression levels of EC signature genes. We observed three different profiles of EC signature genes such as Tek (Tei2), Icam1 (CD54), Ace, Cd34, Cdh5 (CD144), and Pecam1 (CD31) (Supplementary Figure 6). If we consider that genes with < 1000 normalized counts are expressed at a low level, only Flt1 and Kdr were closer to having an appreciable expression level in the D0 and D2 groups. The significant differential expression of Mmp9 on D2 and D4 compared to D0 (Fig. 3) suggests that this enzyme contributed to basement membrane degradation and thus loosened the physical connection between pericytes and ECs, which may have contributed to a reduction of EC contamination in the pericyte fractions from D2. These observations suggest that EC contamination was not significant enough to skew our results. It is also important to point out that pericytes express the EC marker VEGFR1 (Flt1) in an ischemic environment [53, 54], suggesting that these cells may express other EC-related molecules under ischemia as well.

It is important to note that more comprehensive experiments to validate the myogenic potential of pericytes in vivo were out of the current study’s scope, which was designed for a broader initial assessment of the transcriptomic changes in pericytes during regeneration after an ischemic muscle lesion. A detailed demonstration of the myogenic potential of muscle pericytes under ischemia would require lineage tracing experiments using a fluorescence reporter to label pericytes followed by detection of myogenic markers during regeneration after limb ischemia using immunohistology or flow cytometry. Alternatively, single-cell RNA-seq could be performed on isolated pericytes expressing a fluorescent lineage tracer.

### Transcriptomic profile of pericytes demonstrates a shift toward myogenic lineage

In skeletal muscles, pericytes are subdivided into type I and type II cells based on the expression of Nestin. Type I pericytes (Nestin^−^) have the potential to differentiate into fibroblasts or adipocytes [1]; in contrast, type II pericytes (Nestin^+^) are myogenic [1] or neurogenic [13]. The microenvironment may be the main determining factor in the differentiation of pericytes in a living organism. For instance, Birbrair et al. injected type I or II pericytes into two different muscle injury models, representing a regenerative or degenerative environment. Type I pericytes tend to exhibit adipogenic characteristics in the injured muscle, while type II pericytes form myocytes in the regenerating muscle [1]. This concept has also been confirmed with PDGFRα^+^ MSCs [53]. Thus, an understanding of the microenvironment where pericytes develop into myocytes and the changes in their gene expression patterns during this process might be very important in the design of pericyte-based cellular therapies.

According to our transcriptomic analyses, at least a fraction of pericytes may have undergone myogenesis upon ischemia, particularly considering the expression of myosin heavy chains in these cells at the later time points after ischemia (Fig. 5b, e). These findings are consistent with other successful in vivo muscle regeneration experiments performed by transplanting isolated pericytes, likely through myogenesis from pericytes [12, 14, 21–23]. However, we are unaware of other studies that examined gene expression in pericytes isolated based on surface markers at different time points during the course of regeneration after muscle injury. Although our RNA-seq data suggested that a limited portion of the pericyte population committed to myogenesis, other data indicate that pericytes are not multipotent in situ, in contrast to their multipotency in vitro [18]. Clearly, further experiments are required to determine whether pericytes are multipotent in situ, and, if so, what factors are involved in the activation of their multipotency.

Researchers have hypothesized that the migration of pericytes also activates their multipotency [7]. According to our data, upregulation of the expression of *Snail*, whose product is associated with cell motility, may contribute to pericyte migration (Supplementary Figure 4A). The gene expression profile of laminin proteins was altered in pericytes during ischemia (Supplementary Figure 4a and b). Previous studies have shown that the expression of laminin in pericytes regulates their stemness in skeletal muscle [16, 54]. The questions of whether Snail or laminin are the key factors for pericyte stemness and what underlying molecular mechanism may link them to multipotency should be further examined.

### Expression of genes encoding cytokines in pericytes

Cytokine genes expressed in pericytes during muscle regeneration affect several types of cells. Isolated skeletal pericytes secrete many cytokines that regulate endothelial cells and satellite cells [55]. The upregulation of *Igf1* and *Pdgfb* observed in this study may have been important to promote the growth of intrinsic muscle progenitors [55]. Likewise, the upregulation of *Hgf*, *Igf1*, and *Ccl2* observed here after ischemic injury (Supplementary Figure 5a) may have contributed to survival, dispersal, and proliferation of endothelial cells. Further analyses will be necessary to fully understand the biological functions of these cytokines secreted from pericytes in other type of cells during muscle regeneration in vivo.

In our RNA-seq analyses, a significant number of genes involved in leukocyte activation were differentially expressed in pericytes after ischemia, particularly on ischemia day 2 (Supplementary Figure 5b). We also observed a dramatic increase in the number of CD45^+^ hematopoietic cells on ischemia day 2 (Supplementary Figure 5c). Inflammatory regulation is known to be tightly associated with muscle regeneration [32, 56] and angiogenesis [32, 34], and pericytes are known to exhibit an immunomodulatory ability [19, 20, 50, 51]. Notably, pericytes upregulated the expression of signaling molecules that attract inflammatory cells (*Ccl2, Ccl3, Ccl4*, and *Cxcl5*) and molecules that stimulate inflammation (*Il1b* and *Tnf*) at the earliest time point (ischemic day 2) (Supplementary Figure 5a). Beginning on the second day after injury, the expression of these transcripts waned, while the expression of *Igf1* (insulin-like growth factor 1, IGF-1) increased, reaching a maximum at day 7, a time at which *Igf2* (insulin-like growth factor 2, IGF-2) expression was upregulated (Supplementary Figure 5b). Importantly, IGF-1 and IGF-2 have been shown to contribute to the polarization of macrophages toward pro-regenerative and anti-inflammatory phenotypes [57–59], leading to skeletal muscle regeneration [60, 61]. These findings are consistent with a hypothesis originally devised in the context of hepatic injury, according to which pericytes exert a proinflammatory effect on the early stages of tissue injury by promoting the infiltration and activation of inflammatory cells but contribute to macrophage polarization toward pro-regenerative phenotypes later during tissue repair [62]. *Csf1* encodes M-CSF (macrophage colony stimulating factor), which stimulates the proliferation and formation of monocytes/macrophages and increases the blood flow of the ischemic limb by increasing the production of VEGF in the bone marrow [63, 64]. In the present study, we observed that pericytes upregulated *Csf1* expression upon ischemia (Fig. 6d), which suggests that the beneficial effects of overexpression of *Csf1* might be related to one of the communication pathways between pericytes activated during muscle injury and inflammatory cells. Therefore, further studies are warranted to elucidate the crosstalk between pericytes and leukocytes in the context of muscle injury.

## Conclusions

With the increasing number of papers reporting the superiority of using pericytes for cellular therapy [14, 21–24], knowledge of pericyte biology in ischemic tissues is somewhat limited. Here, we report transcriptomic analyses of isolated pericytes isolated from ischemic skeletal muscles. Based on our data, surgery-induced muscle ischemia-triggered pericytes exhibit myogenic potential and may be involved in the modulation of inflammation through the production of paracrine molecules. The data generated in this study may be useful not only to better understand the behavior of pericytes under injury conditions but also to provide a foundation to develop novel gene and cell-based therapies.

## Supplementary Information

**Additional file 1: Supplementary Figure 1.** Event numbers of endothelial cell and pericyte populations. (a-d) Event numbers of (a) endothelial cells, (b) pericytes, (c) type I pericytes and (d) type II pericytes in one cell suspension sample prepared from one ischemic gastrocnemius muscle. *Student*’s t test was used to perform statistical analyses. *, *p* < 0.05; **, *p* < 0.01.

**Additional file 2: Supplementary Figure 2.** Isolation of pericytes (CD45^−^CD31^−^CD146^+^) using FACS. Representative figure of gated pericytes (CD146^+^CD31^−^) (P5) in the CD45^−^ population processed using FACS (upper panel). Then, the purity of the sorted pericytes was analyzed again using the same gating criteria (lower panel).

**Additional file 3: Supplementary Figure 3.** Comparison of enriched KEGG pathways between dynamic profiles. The results of analyses and comparisons of the enriched KEGG pathways in each dynamic profile determined by STEM using ClusterProfiler packages.

**Additional file 4: Supplementary Figure 4.** Heatmaps of the expression levels of genes in cell-cell contact and extracellular matrix categories based on GO terms. Heatmaps present the expression profiles of genes involved in (a) cell junction regulation, (b) ECM regulation, and (c) regulation of cell adhesion that were selected based on the GO tree (see Table 1). The genes mentioned in the text are marked with arrows.

**Additional file 5: Supplementary Figure 5.** Cytokine production in pericytes. Heatmaps present the expression profiles of genes involved in (a) cytokine production and (b) leukocyte activation that were selected based on the GO tree (see Table 1). (c) Quantitation of the CD45^+^ hematopoietic cell population in ischemic muscles. The genes mentioned in the text are marked with arrows.

**Additional file 6: Supplementary figure 6.** Expression of signature genes of endothelial cells**.** Three expression profiles of endothelial cells were found: (a) The expression levels of Tek (Tei2) and Icam1 (CD54), (b) The expression levels of Ace and Cd34, (c) The expression level of Cdh5 (CD144) and Pecam1 (CD31). The results of the statistical analysis shown are the FDR calculated using DEseq2. *, FDR < 0.05; **, FDR < 0.01.

## Data Availability

All data generated and/or analyzed during this study are included in this published article.

## Abbreviations

CLI: Critical limb ischemia
DE: Differentially expressed
ECM: Extracellular matrix
FACS: Fluorescence activated cell sorting
FMO: Fluorescence minus one
GO: Gene Ontology
KEGG: Kyoto Encyclopedia of Genes and Genomes
NG2: Neuron glia antigen-2
NGFR: Nerve growth factor receptor
MMP: Matrix metallopeptidases
MSC: Mesenchymal stem/stromal cells
PAD: Peripheral arterial disease
PDGFRb: Platelet-derived growth factor receptor beta
PDGFb: Platelet-derived growth factor subunit B
VEGF: Vascular endothelial growth factor
