# Supplementary figures and images for: Analyses of the pericyte transcriptome in ischemic skeletal muscles
Source: Stem Cell Res Ther. 2021 Mar 16;12:183. doi: 10.1186/s13287-021-02247-3 (PMC7962292; doi:10.1186/s13287-021-02247-3)

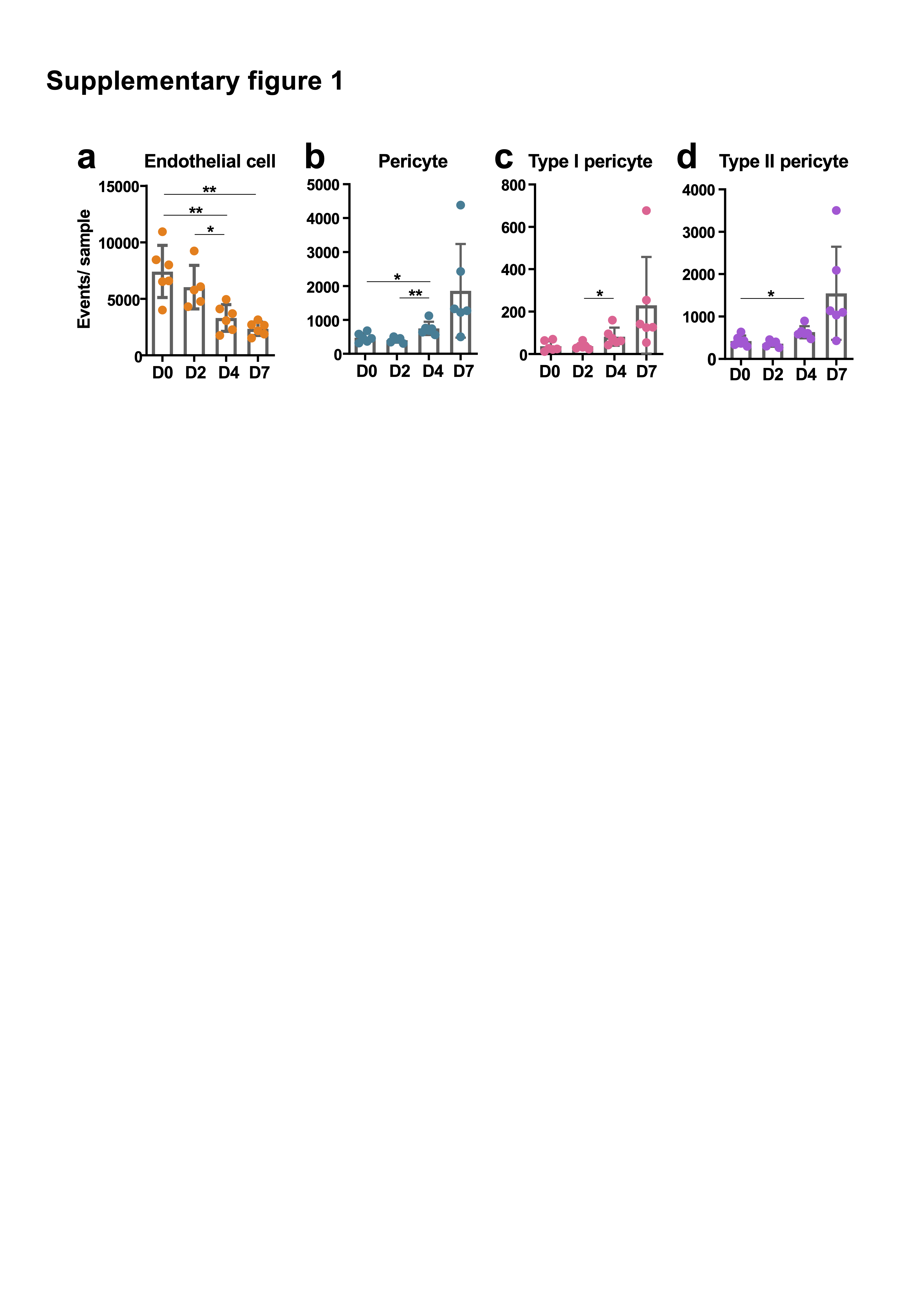

Supplement: Supplementary file 1 — Additional file 1: Supplementary Figure 1. Event numbers of endothelial cell and pericyte populations. (a-d) Event numbers of (a) endothelial cells, (b) pericytes, (c) type I pericytes and (d) type II pericytes in one cell suspension sample prepared from one ischemic gastrocnemius muscle. Student’s t test was used to perform statistical analyses. *, p < 0.05; **, p < 0.01. [file 13287_2021_2247_MOESM1_ESM.tiff]

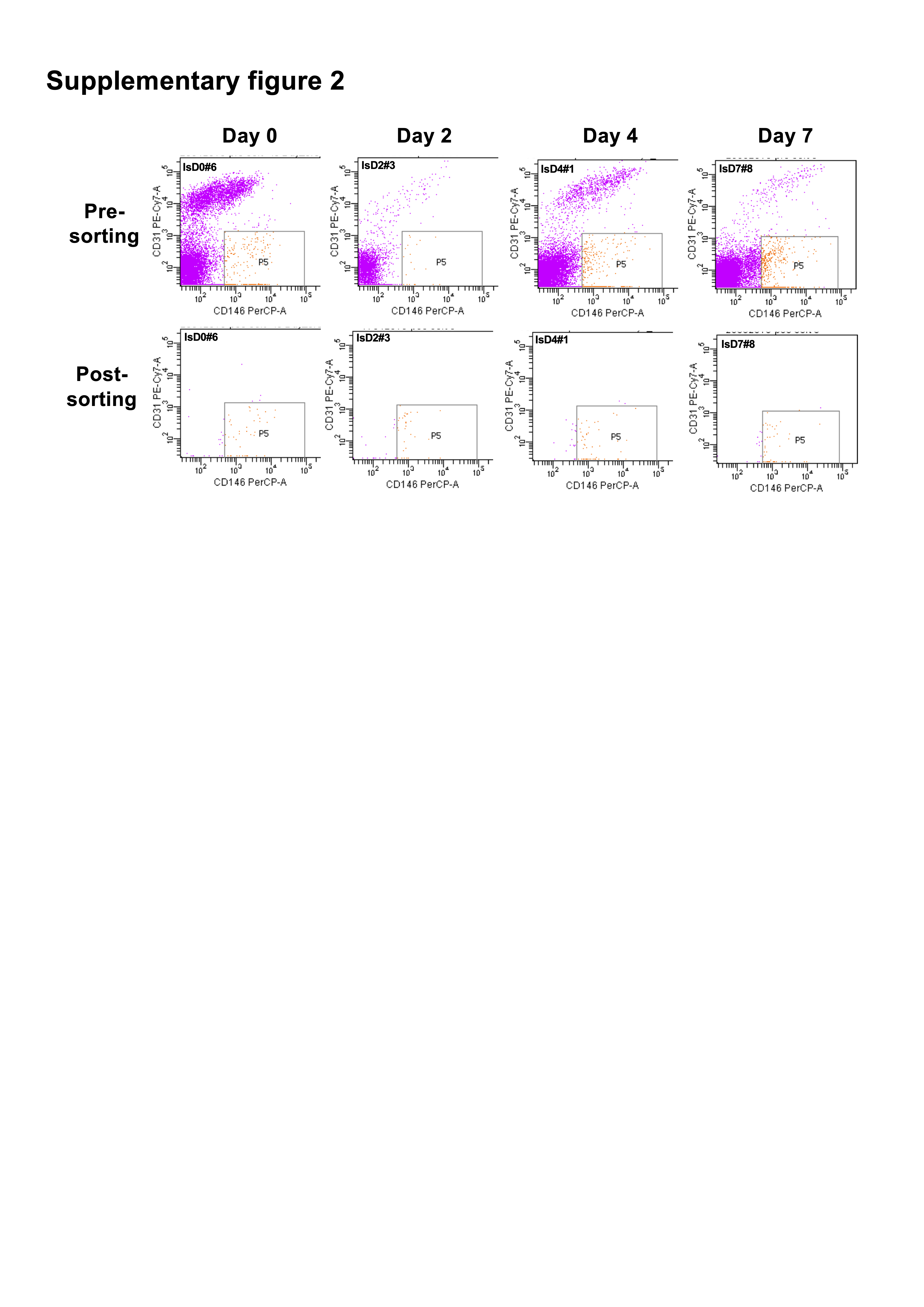

Supplement: Supplementary file 2 — Additional file 2: Supplementary Figure 2. Isolation of pericytes (CD45−CD31−CD146+) using FACS. Representative figure of gated pericytes (CD146+CD31−) (P5) in the CD45− population processed using FACS (upper panel). Then, the purity of the sorted pericytes was analyzed again using the same gating criteria (lower panel). [file 13287_2021_2247_MOESM2_ESM.tiff]

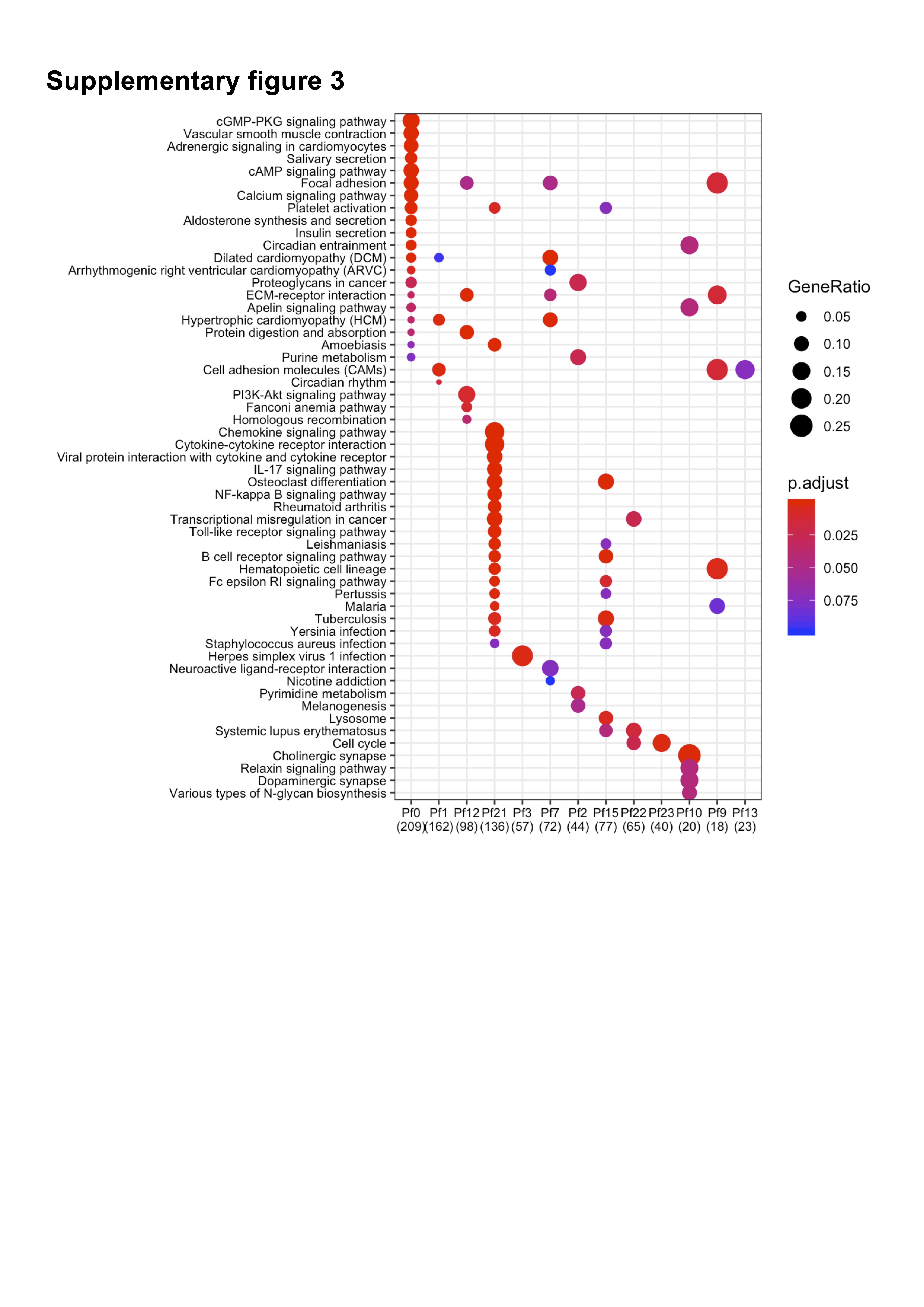

Supplement: Supplementary file 3 — Additional file 3: Supplementary Figure 3. Comparison of enriched KEGG pathways between dynamic profiles. The results of analyses and comparisons of the enriched KEGG pathways in each dynamic profile determined by STEM using ClusterProfiler packages. [file 13287_2021_2247_MOESM3_ESM.tiff]

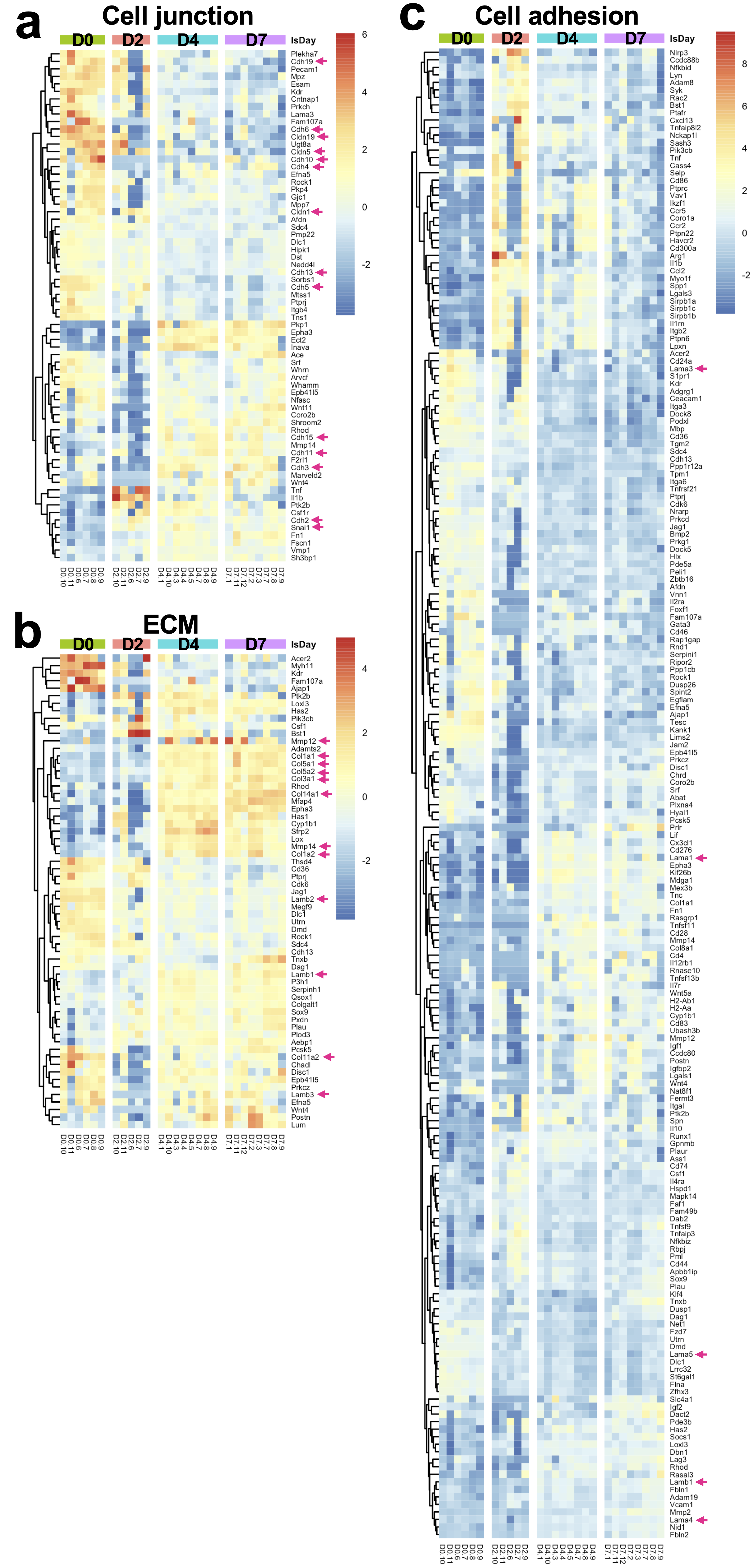

Supplement: Supplementary file 4 — Additional file 4: Supplementary Figure 4. Heatmaps of the expression levels of genes in cell-cell contact and extracellular matrix categories based on GO terms. Heatmaps present the expression profiles of genes involved in (a) cell junction regulation, (b) ECM regulation, and (c) regulation of cell adhesion that were selected based on the GO tree (see Table 1). The genes mentioned in the text are marked with arrows. [file 13287_2021_2247_MOESM4_ESM.tiff]

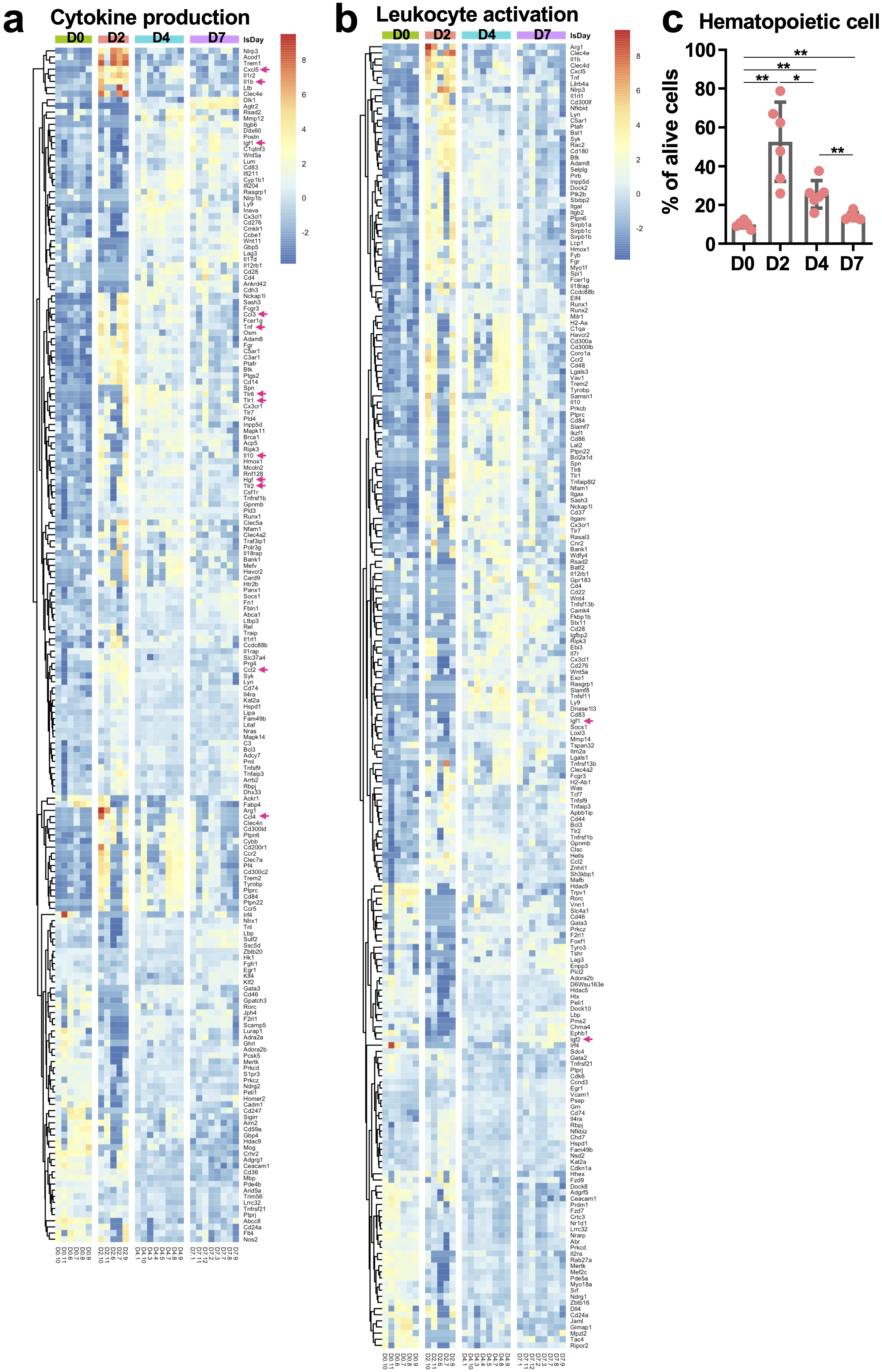

Supplement: Supplementary file 5 — Additional file 5: Supplementary Figure 5. Cytokine production in pericytes. Heatmaps present the expression profiles of genes involved in (a) cytokine production and (b) leukocyte activation that were selected based on the GO tree (see Table 1). (c) Quantitation of the CD45+ hematopoietic cell population in ischemic muscles. The genes mentioned in the text are marked with arrows. [file 13287_2021_2247_MOESM5_ESM.tiff]

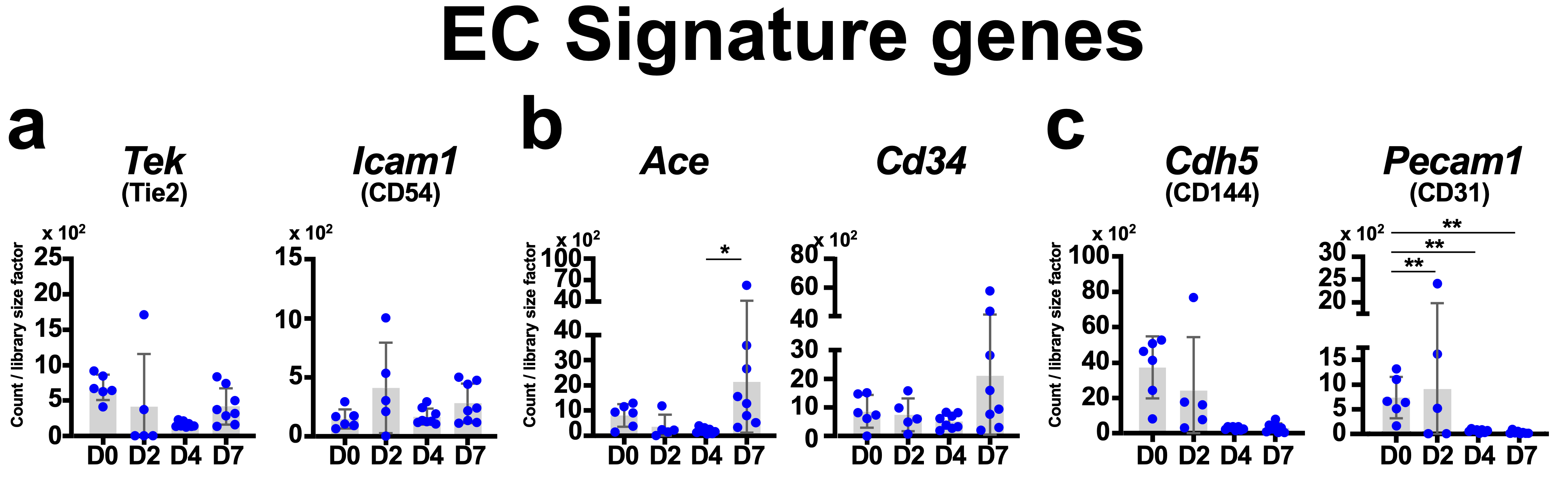

Supplement: Supplementary file 6 — Additional file 6: Supplementary figure 6. Expression of signature genes of endothelial cells. Three expression profiles of endothelial cells were found: (a) The expression levels of Tek (Tei2) and Icam1 (CD54), (b) The expression levels of Ace and Cd34, (c) The expression level of Cdh5 (CD144) and Pecam1 (CD31). The results of the statistical analysis shown are the FDR calculated using DEseq2. *, FDR < 0.05; **, FDR < 0.01. [file 13287_2021_2247_MOESM6_ESM.tiff]
